# 2,6-Di-*tert*-butyl-4-(3-chloro-2-hy­droxy­prop­yl)phenol

**DOI:** 10.1107/S1600536811008592

**Published:** 2011-03-12

**Authors:** Ayten R. Asgarova, Abel M. Maharramov, Ali N. Khalilov, Atash V. Gurbanov, Seik Weng Ng

**Affiliations:** a Department of Organic Chemistry, Baku State University, Baku, Azerbaijan; b Department of Chemistry, University of Malaya, 50603 Kuala Lumpur, Malaysia

## Abstract

In the title 2-propanol derivative, C_17_H_27_ClO_2_, the two *tert*-butyl groups both have one methyl C atom lying in the plane of the aromatic ring. In the crystal, the phenol group forms a hydrogen bond to the hy­droxy O atom belonging to the alkyl substituent of an adjacent mol­ecule, forming a chain along the *ac* diagonal. The Cl atom is disordered over two positions in a 0.73 (4):0.27 (4) ratio.

## Related literature

For the synthesis: see: Krysin *et al.* (2010 ▶).
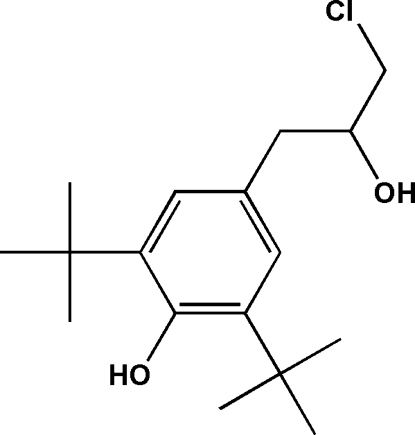


## Experimental

### 

#### Crystal data

C_17_H_27_ClO_2_
*M* *_r_* = 298.84
Monoclinic, 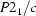

*a* = 5.9536 (3) Å
*b* = 19.4819 (9) Å
*c* = 14.4310 (7) Å
β = 96.798 (1)°
*V* = 1662.05 (14) Å^3^
*Z* = 4
Mo *K*α radiation
μ = 0.23 mm^−1^
*T* = 100 K
0.30 × 0.30 × 0.30 mm

#### Data collection

Bruker APEXII diffractometer
Absorption correction: multi-scan (*SADABS*; Sheldrick, 1996 ▶) *T*_min_ = 0.934, *T*_max_ = 0.934
17600 measured reflections
3819 independent reflections
3374 reflections with *I* > 2σ(*I*)
*R*_int_ = 0.035

#### Refinement

*R*[*F* ^2^ > 2σ(*F* ^2^)] = 0.049
*wR*(*F* ^2^) = 0.131
*S* = 1.12
3819 reflections
193 parameters
1 restraint
H-atom parameters constrained
Δρ_max_ = 0.59 e Å^−3^
Δρ_min_ = −0.36 e Å^−3^

### 

Data collection: *APEX2* (Bruker, 2005 ▶); cell refinement: *SAINT* (Bruker, 2005 ▶); data reduction: *SAINT*; program(s) used to solve structure: *SHELXS97* (Sheldrick, 2008 ▶); program(s) used to refine structure: *SHELXL97* (Sheldrick, 2008 ▶); molecular graphics: *X-SEED* (Barbour, 2001 ▶); software used to prepare material for publication: *publCIF* (Westrip, 2010 ▶).

## Supplementary Material

Crystal structure: contains datablocks global, I. DOI: 10.1107/S1600536811008592/xu5167sup1.cif

Structure factors: contains datablocks I. DOI: 10.1107/S1600536811008592/xu5167Isup2.hkl

Additional supplementary materials:  crystallographic information; 3D view; checkCIF report

## Figures and Tables

**Table 1:** Hydrogen-bond geometry (Å, °)

*D*—H⋯*A*	*D*—H	H⋯*A*	*D*⋯*A*	*D*—H⋯*A*
O2—H2⋯O1^i^	0.84	2.31	2.956 (2)	134

Symmetry code: (i) 
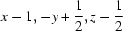
.

**Fig. 1.**
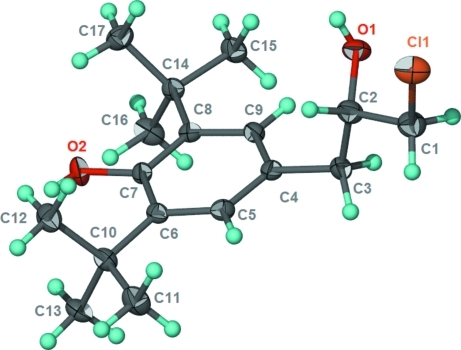
Thermal ellipsoid plot (Barbour, 2001) of C17H27ClO2 at the 70% probability level; hydrogen atoms are drawn as spheres of arbitrary radius. The disorder in the chlorine atom is not shown.

**Fig. 2.**
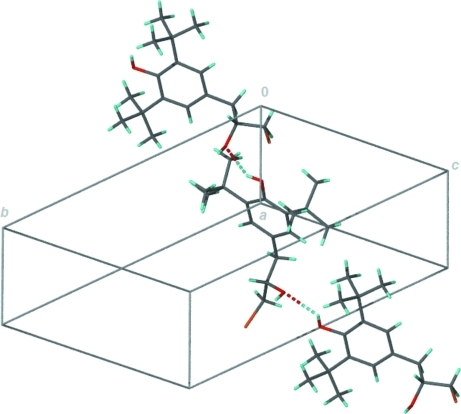
Hydrogen-bonded chain motif.

## supplementary crystallographic information

### Comment

The chlorohydrin unit (*i.e.*, an alkyl chain having a chlorine atom and a hydroxy group on adjacent carbons) is an important unit in compounds used for the treatment of protozoal and bacterial infections; and chlorohydrin-based compounds are important intermediates in the synthesis of some HIV protease inhibitors. The di-*tert*-butyl phenol unit is also an important component of medicinal compounds. The two units are assembled in the title compound (Scheme I).

The compound can be further transformed; in fact, replacing the chlorine atom by a diisopropylamino group furnishes a 2:1 co-crystal with succinic acid that has been patented for its antiarrhythmic and antihypertensive activities (Krysin *et al.*, 2010).

The two *tert*-butyl groups of C_17_H_27_ClO_2_ both have one methyl C lying in the plane of the aromatic ring (Fig. 1). The phenolic group forms a hydrogen bond to the hydroxy O atom belonging to the alkyl substituent of an adjacent molecule to form a chain along the *a*–*c* diagonal of the monoclinic unit cell (Fig. 2).

### Experimental

The compound was prepared by using a procedure reported in the patent literature (Krysin *et al.*, 2010), and colorless crystals was obtained upon recrystallization from ethanol.

### Refinement

Carbon-bound H-atoms were placed in calculated positions [C–H 0.93 to 0.97 Å; *U*(H) 1.2 to 1.5*U*(C)] and were included in the refinement in the riding model approximation. The hydroxy H-atoms were similarly treated (O–H 0.84 Å) and their temperature factors tied by a factor of 1.5.

The chlorine atom is disordered over two positions; the C–Cl pair of distances were restrained to within Å of each other. The disordered refined to a 73 (4): 27 ratio. The thermal ellipsoid of the minor component is somewhat elongated; however, no restraints were imposed to render it to be less elongated.

### Crystal data

**Table:**

C_17_H_27_ClO_2_	*F*(000) = 648
*M**_r_* = 298.84	*D*_x_ = 1.194 Mg m^−^^3^
Monoclinic, *P*2_1_/*c*	Mo *K*α radiation, λ = 0.71073 Å
Hall symbol: -P 2ybc	Cell parameters from 6280 reflections
*a* = 5.9536 (3) Å	θ = 2.5–28.3°
*b* = 19.4819 (9) Å	µ = 0.23 mm^−^^1^
*c* = 14.4310 (7) Å	*T* = 100 K
β = 96.798 (1)°	Prism, colorless
*V* = 1662.05 (14) Å^3^	0.30 × 0.30 × 0.30 mm
*Z* = 4	

### Data collection

**Table:**

Bruker APEXII diffractometer	3819 independent reflections
Radiation source: fine-focus sealed tube	3374 reflections with *I* > 2σ(*I*)
graphite	*R*_int_ = 0.035
φ and ω scans	θ_max_ = 27.5°, θ_min_ = 1.8°
Absorption correction: multi-scan (*SADABS*; Sheldrick, 1996)	*h* = −7→7
*T*_min_ = 0.934, *T*_max_ = 0.934	*k* = −25→25
17600 measured reflections	*l* = −18→18

### Refinement

**Table:**

Refinement on *F*^2^	Primary atom site location: structure-invariant direct methods
Least-squares matrix: full	Secondary atom site location: difference Fourier map
*R*[*F*^2^ > 2σ(*F*^2^)] = 0.049	Hydrogen site location: inferred from neighbouring sites
*wR*(*F*^2^) = 0.131	H-atom parameters constrained
*S* = 1.12	*w* = 1/[σ^2^(*F*_o_^2^) + (0.057*P*)^2^ + 1.2061*P*] where *P* = (*F*_o_^2^ + 2*F*_c_^2^)/3
3819 reflections	(Δ/σ)_max_ = 0.001
193 parameters	Δρ_max_ = 0.59 e Å^−^^3^
1 restraint	Δρ_min_ = −0.36 e Å^−^^3^

### Fractional atomic coordinates and isotropic or equivalent isotropic displacement parameters (Å^2^)

**Table:**

	*x*	*y*	*z*	*U*_iso_*/*U*_eq_	Occ. (<1)
Cl1	1.2917 (5)	0.47575 (14)	0.5791 (3)	0.0266 (6)	0.73 (4)
Cl1'	1.311 (2)	0.4728 (6)	0.5926 (16)	0.048 (2)	0.27 (4)
O1	1.1454 (2)	0.31683 (6)	0.56395 (9)	0.0212 (3)	
H1	1.2420	0.3195	0.5261	0.032*	
O2	0.3696 (2)	0.18279 (6)	0.25797 (9)	0.0199 (3)	
H2	0.3138	0.2071	0.2130	0.030*	
C1	1.0528 (3)	0.43104 (10)	0.61378 (14)	0.0235 (4)	
H1A	1.0903	0.4130	0.6779	0.028*	0.73 (4)
H1B	0.9240	0.4631	0.6139	0.028*	0.73 (4)
H1'A	1.0701	0.4134	0.6786	0.028*	0.27 (4)
H1'B	0.9292	0.4653	0.6082	0.028*	0.27 (4)
C2	0.9867 (3)	0.37230 (9)	0.54759 (13)	0.0202 (4)	
H2A	0.9834	0.3886	0.4817	0.024*	
C3	0.7524 (3)	0.34483 (9)	0.56317 (12)	0.0190 (4)	
H3A	0.7664	0.3169	0.6209	0.023*	
H3B	0.6514	0.3840	0.5721	0.023*	
C4	0.6473 (3)	0.30175 (9)	0.48281 (12)	0.0159 (3)	
C5	0.5064 (3)	0.33223 (9)	0.41043 (12)	0.0159 (3)	
H5	0.4776	0.3801	0.4134	0.019*	
C6	0.4059 (3)	0.29514 (8)	0.33367 (11)	0.0144 (3)	
C7	0.4559 (3)	0.22451 (9)	0.33061 (11)	0.0146 (3)	
C8	0.5969 (3)	0.19141 (8)	0.40264 (11)	0.0145 (3)	
C9	0.6889 (3)	0.23165 (9)	0.47749 (12)	0.0155 (3)	
H9	0.7837	0.2104	0.5268	0.019*	
C10	0.2464 (3)	0.33124 (9)	0.25678 (12)	0.0169 (3)	
C11	0.2100 (3)	0.40719 (9)	0.27950 (14)	0.0244 (4)	
H11A	0.3558	0.4311	0.2862	0.037*	
H11B	0.1420	0.4107	0.3379	0.037*	
H11C	0.1089	0.4282	0.2289	0.037*	
C12	0.3501 (3)	0.33046 (10)	0.16400 (13)	0.0225 (4)	
H12A	0.4996	0.3522	0.1729	0.034*	
H12B	0.2516	0.3558	0.1166	0.034*	
H12C	0.3653	0.2829	0.1434	0.034*	
C13	0.0101 (3)	0.29757 (10)	0.24768 (13)	0.0211 (4)	
H13A	−0.0494	0.2991	0.3081	0.032*	
H13B	0.0218	0.2497	0.2279	0.032*	
H13C	−0.0922	0.3226	0.2013	0.032*	
C14	0.6512 (3)	0.11431 (8)	0.39914 (12)	0.0158 (3)	
C15	0.8079 (3)	0.09121 (9)	0.48583 (13)	0.0215 (4)	
H15A	0.9483	0.1179	0.4907	0.032*	
H15B	0.8430	0.0423	0.4803	0.032*	
H15C	0.7324	0.0986	0.5417	0.032*	
C16	0.4356 (3)	0.07064 (9)	0.39586 (14)	0.0234 (4)	
H16A	0.3307	0.0835	0.3411	0.035*	
H16B	0.3637	0.0785	0.4526	0.035*	
H16C	0.4749	0.0220	0.3917	0.035*	
C17	0.7752 (3)	0.09881 (9)	0.31401 (13)	0.0216 (4)	
H17A	0.6804	0.1128	0.2570	0.032*	
H17B	0.8062	0.0495	0.3114	0.032*	
H17C	0.9182	0.1242	0.3193	0.032*	

### Atomic displacement parameters (Å^2^)

**Table:**

	*U*^11^	*U*^22^	*U*^33^	*U*^12^	*U*^13^	*U*^23^
Cl1	0.0251 (8)	0.0174 (9)	0.0380 (11)	−0.0002 (7)	0.0068 (6)	0.0062 (11)
Cl1'	0.022 (2)	0.042 (4)	0.078 (5)	−0.016 (2)	0.001 (3)	−0.034 (3)
O1	0.0165 (6)	0.0191 (6)	0.0277 (7)	0.0035 (5)	0.0020 (5)	−0.0010 (5)
O2	0.0264 (7)	0.0159 (6)	0.0154 (6)	0.0009 (5)	−0.0061 (5)	−0.0014 (5)
C1	0.0196 (9)	0.0227 (9)	0.0280 (10)	−0.0023 (7)	0.0022 (7)	−0.0025 (7)
C2	0.0180 (8)	0.0182 (8)	0.0238 (9)	0.0019 (7)	−0.0002 (7)	−0.0011 (7)
C3	0.0171 (8)	0.0202 (8)	0.0186 (8)	0.0021 (7)	−0.0020 (6)	−0.0044 (7)
C4	0.0122 (7)	0.0189 (8)	0.0167 (8)	−0.0006 (6)	0.0022 (6)	−0.0027 (6)
C5	0.0154 (8)	0.0137 (7)	0.0189 (8)	0.0005 (6)	0.0029 (6)	−0.0001 (6)
C6	0.0128 (7)	0.0158 (8)	0.0145 (8)	0.0002 (6)	0.0017 (6)	0.0022 (6)
C7	0.0139 (7)	0.0165 (8)	0.0133 (8)	−0.0011 (6)	0.0015 (6)	−0.0016 (6)
C8	0.0132 (7)	0.0146 (8)	0.0160 (8)	0.0012 (6)	0.0030 (6)	0.0009 (6)
C9	0.0132 (7)	0.0189 (8)	0.0141 (8)	0.0013 (6)	0.0008 (6)	0.0012 (6)
C10	0.0157 (8)	0.0163 (8)	0.0180 (8)	0.0003 (6)	−0.0011 (6)	0.0022 (6)
C11	0.0277 (10)	0.0159 (8)	0.0276 (10)	0.0041 (7)	−0.0051 (8)	0.0024 (7)
C12	0.0237 (9)	0.0257 (9)	0.0176 (9)	−0.0010 (7)	0.0007 (7)	0.0059 (7)
C13	0.0142 (8)	0.0238 (9)	0.0242 (9)	0.0003 (7)	−0.0018 (7)	0.0020 (7)
C14	0.0171 (8)	0.0138 (8)	0.0162 (8)	0.0014 (6)	0.0006 (6)	0.0013 (6)
C15	0.0228 (9)	0.0182 (8)	0.0222 (9)	0.0051 (7)	−0.0023 (7)	0.0027 (7)
C16	0.0205 (9)	0.0191 (9)	0.0298 (10)	−0.0025 (7)	0.0001 (7)	0.0053 (7)
C17	0.0256 (9)	0.0173 (8)	0.0226 (9)	0.0044 (7)	0.0058 (7)	−0.0006 (7)

### Geometric parameters (Å, °)

**Table:**

Cl1—C1	1.788 (3)	C9—H9	0.9500
Cl1'—C1	1.795 (6)	C10—C11	1.537 (2)
O1—C2	1.437 (2)	C10—C12	1.539 (3)
O1—H1	0.8400	C10—C13	1.544 (2)
O2—C7	1.377 (2)	C11—H11A	0.9800
O2—H2	0.8400	C11—H11B	0.9800
C1—C2	1.513 (3)	C11—H11C	0.9800
C1—H1A	0.9900	C12—H12A	0.9800
C1—H1B	0.9900	C12—H12B	0.9800
C1—H1'A	0.9900	C12—H12C	0.9800
C1—H1'B	0.9900	C13—H13A	0.9800
C2—C3	1.535 (2)	C13—H13B	0.9800
C2—H2A	1.0000	C13—H13C	0.9800
C3—C4	1.506 (2)	C14—C17	1.536 (2)
C3—H3A	0.9900	C14—C16	1.536 (2)
C3—H3B	0.9900	C14—C15	1.537 (2)
C4—C9	1.392 (2)	C15—H15A	0.9800
C4—C5	1.393 (2)	C15—H15B	0.9800
C5—C6	1.396 (2)	C15—H15C	0.9800
C5—H5	0.9500	C16—H16A	0.9800
C6—C7	1.410 (2)	C16—H16B	0.9800
C6—C10	1.542 (2)	C16—H16C	0.9800
C7—C8	1.412 (2)	C17—H17A	0.9800
C8—C9	1.393 (2)	C17—H17B	0.9800
C8—C14	1.539 (2)	C17—H17C	0.9800
			
C2—O1—H1	109.5	C11—C10—C6	112.10 (14)
C7—O2—H2	109.5	C12—C10—C6	110.19 (14)
C2—C1—Cl1	110.38 (18)	C11—C10—C13	106.08 (14)
C2—C1—Cl1'	113.5 (5)	C12—C10—C13	112.16 (15)
C2—C1—H1A	109.6	C6—C10—C13	110.17 (14)
Cl1—C1—H1A	109.6	C10—C11—H11A	109.5
Cl1'—C1—H1A	102.5	C10—C11—H11B	109.5
C2—C1—H1B	109.6	H11A—C11—H11B	109.5
Cl1—C1—H1B	109.6	C10—C11—H11C	109.5
Cl1'—C1—H1B	113.1	H11A—C11—H11C	109.5
H1A—C1—H1B	108.1	H11B—C11—H11C	109.5
C2—C1—H1'A	108.9	C10—C12—H12A	109.5
Cl1—C1—H1'A	115.9	C10—C12—H12B	109.5
Cl1'—C1—H1'A	108.9	H12A—C12—H12B	109.5
C2—C1—H1'B	108.9	C10—C12—H12C	109.5
Cl1'—C1—H1'B	108.9	H12A—C12—H12C	109.5
H1'A—C1—H1'B	107.7	H12B—C12—H12C	109.5
O1—C2—C1	110.37 (15)	C10—C13—H13A	109.5
O1—C2—C3	107.78 (14)	C10—C13—H13B	109.5
C1—C2—C3	110.14 (15)	H13A—C13—H13B	109.5
O1—C2—H2A	109.5	C10—C13—H13C	109.5
C1—C2—H2A	109.5	H13A—C13—H13C	109.5
C3—C2—H2A	109.5	H13B—C13—H13C	109.5
C4—C3—C2	112.54 (14)	C17—C14—C16	110.21 (15)
C4—C3—H3A	109.1	C17—C14—C8	109.93 (13)
C2—C3—H3A	109.1	C16—C14—C8	111.33 (14)
C4—C3—H3B	109.1	C17—C14—C15	106.87 (14)
C2—C3—H3B	109.1	C16—C14—C15	106.72 (14)
H3A—C3—H3B	107.8	C8—C14—C15	111.65 (14)
C9—C4—C5	118.18 (15)	C14—C15—H15A	109.5
C9—C4—C3	121.94 (15)	C14—C15—H15B	109.5
C5—C4—C3	119.88 (15)	H15A—C15—H15B	109.5
C6—C5—C4	122.53 (15)	C14—C15—H15C	109.5
C6—C5—H5	118.7	H15A—C15—H15C	109.5
C4—C5—H5	118.7	H15B—C15—H15C	109.5
C5—C6—C7	117.23 (15)	C14—C16—H16A	109.5
C5—C6—C10	120.29 (15)	C14—C16—H16B	109.5
C7—C6—C10	122.48 (15)	H16A—C16—H16B	109.5
O2—C7—C6	122.59 (15)	C14—C16—H16C	109.5
O2—C7—C8	115.26 (14)	H16A—C16—H16C	109.5
C6—C7—C8	122.14 (15)	H16B—C16—H16C	109.5
C9—C8—C7	117.30 (15)	C14—C17—H17A	109.5
C9—C8—C14	120.65 (14)	C14—C17—H17B	109.5
C7—C8—C14	122.04 (15)	H17A—C17—H17B	109.5
C4—C9—C8	122.60 (15)	C14—C17—H17C	109.5
C4—C9—H9	118.7	H17A—C17—H17C	109.5
C8—C9—H9	118.7	H17B—C17—H17C	109.5
C11—C10—C12	106.04 (15)		

### Hydrogen-bond geometry (Å, °)

**Table:**

*D*—H···*A*	*D*—H	H···*A*	*D*···*A*	*D*—H···*A*
O2—H2···O1^i^	0.84	2.31	2.956 (2)	134

Symmetry codes: (i) *x*−1, −*y*+1/2, *z*−1/2.
